# Light as a Caustic

**Published:** 1893-01

**Authors:** 


### Light as a Caustic.

In a very interesting article by Dr. Arnold in the *Pacific Medical Journal* (November) there are some striking passages concerning the caustic effects of light, with and without its accompanying heat-rays. He says:

"For practical purposes the heat-rays can be filtered out of a beam of sunlight with a flat cell filled with a strong solution of alum, the most eligible athermanous material; and concentration of the light may be increased about twenty-fold before the painful effects of heat concentration are experienced. A common whisky flask, free from flaws, filled with alum solution, and a lens, such as is sold for examining photographs, will serve for all minor purposes. This degree of concentration I have frequently employed on boils, warts, ring-worms and the like, with instructive results; although they are too inexact experiments, from the trifling nature of the cases and from the absence of control, to describe them in detail.

I have secured distinct escharotic effects from a greater degree of concentration, although cosmoline would barely melt at the focus of the lens, a temperature of about 125° F.; and that effect was attained in two cases with a slight pricking sensation hardly amounting to pain, although the entire thickness of skin was reduced to a whitish, pulpy mass. This agrees with an observation already referred to, and with Dr. Thayer's experience, in which the simple burning glass was used without anæsthetizing the patient. The pain after a time was not complained of, notwithstanding the high temperature developed at the focus of the lens. He records, as the principal objection to the treatment the unpleasant appearance of the operation and the odor and the smoke from the burning flesh.

Since 120° F. is about the limit of usual toleration of moist heat, and since 145° dry heat produces nearly intolerable pain in one minute, and in ten minutes causes quite active hyperæmia, it is plain that concentrated light, minus its grosser heat, is both anæsthetic and escharotic, the last in a mild degree.

In considering the former property, some allowance must be made for the effect of dessication, which, although hindered somewhat in this case by the transudation of serum, has an admitted effect. The latter property manifests itself in the severe blistering of the skin at elevations above the snow line and in small boats on the water in the tropics, being much severer in the early and late hours of the day, from the oblique position of the sun and the greater consequent reflection of the rays from the surface of the water.—*Maryland Medical Journal.*

